# Combination of S100B and procalcitonin improves prognostic performance compared to either alone in patients with cardiac arrest: A prospective observational study

**DOI:** 10.1097/MD.0000000000014496

**Published:** 2019-02-08

**Authors:** Jae Ho Jang, Won Bin Park, Yong Su Lim, Jea Yeon Choi, Jin Seong Cho, Jae-Hyug Woo, Woo Sung Choi, Hyuk Jun Yang, Sung Youl Hyun

**Affiliations:** a Department of Emergency Medicine, Gachon University Gil Medical Center; b Department of Emergency Medicine; c Department of Traumatology, Gil Medical Center, Gachon University College of Medicine, Incheon, South Korea.

**Keywords:** biomarkers, cardiac arrest, outcome, pro-calcitonin, S100b Protein

## Abstract

This study aimed to determine whether the combination of procalcitonin (PCT) and S100B improves prognostic performance compared to either alone in cardiac arrest (CA) patients treated with targeted temperature management (TTM).

We performed a prospective cohort study of CA patients treated with TTM. PCT and S100B levels were obtained at 0, 24, 48, and 72 hours after return of spontaneous circulation. The prognostic performance was analyzed using each marker and the combination of the 2 markers for predicting poor neurological outcome at 3 months and mortality at 14 days and 3 months.

A total of 97 patients were enrolled, of which 67 (69.1%) had poor neurological outcome. S100B showed a better prognostic performance (area under the curve [AUC], 0.934; sensitivity, 77.6%; and specificity, 100%) than PCT (AUC, 0.861; sensitivity, 70.2%; and specificity, 83.3%) with the highest prognostic value at 24 hours. The combination of 24-hour PCT and S100B values (S100B ≥0.2 μg/L or PCT ≥6.6 ng/mL) improved sensitivity (85.07%) compared with S100B alone. In multivariate analysis, PCT was associated with mortality at 14 days (odds ratio [OR]: 1.064, 95% confidence interval [CI]: 1.014–1.118), whereas S100B was associated with neurological outcomes at 3 months (OR: 9.849, 95% CI: 2.089–46.431).

The combination of PCT and S100B improved prognostic performance compared to the use of either biomarker alone in CA patient treated with TTM. Further studies that will identify the optimal cutoff values for these biomarkers must be conducted.

## 1 Introduction

Early and accurate prediction of outcome is one of the most important aspects of post-cardiac arrest (CA) care, which can help select patients for intensive care, avoid inappropriate treatment, and provide information for relatives.^[1]^ Although various approaches have been used for prediction outcome, early prognostication is difficult and challenging. Recently, a multimodal approach for prognostication is recommended^[2]^ because there is no single accurate predictor. However, studies on the efficacy of the specific combinations of prognostication tools are limited.

For the prognostication of post-CA patients, various biomarkers have been investigated because blood biomarkers have the advantages over other tools, such as quantitative results, and likely independent from the effects of sedatives.^[1]^ Most studied biomarkers for the prognostication of post-CA patients include neuron-specific enolase (NSE) and S100B protein,^[3–11]^ which were correlated with the extent of anoxic–ischemic neurological injury from CA and neurological outcomes. Although brain injury is the major cause of death after return of spontaneous circulation (ROSC) in post-CA patients, significant proportion of these patients died after hospitalization because of non-brain injury-related causes, such as cardiovascular shock and multiple organ dysfunction (MOD).^[12–14]^ Therefore, the use of a brain-specific biomarker alone for prognostication after CA is insufficient.^[15]^

Recent studies^[4,16,17]^ have shown that procalcitonin (PCT), an inflammatory marker, was associated with the severity of post-CA syndrome (PCAS) and unfavorable outcomes, and it is a potential marker for the early identification of patients who are at high risk for PCAS-related circulatory failure, MOD, and death. Therefore, PCT may be a candidate biomarker that can be used in the multimodal approach for prognostication after CA.^[18]^

We hypothesized that PCT and S100B levels in the blood may be utilized for the prognostication of post-CA patients based on their biochemical characteristics, and the combination of PCT, which is correlated with extra-cerebral organ dysfunction and systemic inflammatory response, and S100B protein, which is brain injury specific, can improve prognostic performance.

Thus, this study aimed to evaluate the prognostic performance of serum PCT and S100B in comatose CA survivors treated with targeted temperature management **(**TTM). Moreover, whether the combination of both biomarkers improves prognostic performance compared to the use of either biomarker alone was investigated.

## 2 Methods

### 2.1 Study population and setting

We conducted a single-center, prospective observational cohort study between June 2010 and May 2014 at a tertiary hospital located in metropolitan city. This study was approved by the institutional review board of our hospital (GIRBA2313), adult patients (aged >18 years) who were successfully resuscitated and achieved ROSC after non-traumatic out of hospital CA (OHCA) and then received TTM at 33°C were enrolled in this study. Patients with possible causes of coma other than CA (head injury, poisoning, or cerebrovascular accident) and those with severe neurological disorder or stroke, end-stage non-cardiac disease, severe disability (Glasgow-Pittsburgh cerebral performance category, CPC ≥3), or end-stage renal or liver disease were excluded. In addition, patients who died within 24 hours after hospital admission were not included. Demographic data were obtained from a prospective registry database of all patients treated in the emergency room after CA and from the pre- and in- hospital medical records.

### 2.2 Blood sampling and measurements

Blood samples used for the measurement of PCT and S100B were collected at baseline (BL), 24, 48, and 72 hours after ROSC. Blood samples were allowed to clot for 30 minutes at 4°C and were centrifuged at 3000 rpm for 10 minutes at 4°C, and the supernatant was stored at −80°C until analysis.

PCT values were measured using an enzyme-linked fluorescent immunoassay (bioMerieux VIDAS B.R.A.H.M.S. PCT, France) on the Cobas e601 analyzer (Roche, Basel, Switzerland), and the detection limit was 0.05 ng/mL. The S100B levels were measured with the immunoluminometric assay method (Elecsys 2010, Roche Diagnostics, Mannheim, Germany) using the Cobas e601 analyzer, and the assay detection limit was 0.005 μg/L.

### 2.3 Assessment of clinical outcomes and severity

At 3 months after CA, we assessed the clinical neurological outcome of patients using the Glasgow-Pittsburgh cerebral performance category (CPC).^[19]^ In addition, at 14 days and 3 months after CA, we assessed deaths to investigate the association with mortality because previously a large cohort study reported that only 5% of post-CA patients died beyond 14 days.^[14]^ The patients were classified into 2 groups based on neurological outcomes: the good neurological outcome group (CPC 1 and CPC 2) and the poor neurological outcome group (CPC 3–CPC 5). To quantify the severity of PCAS, acute physiology and chronic health evaluation (APACHE) II scores^[20]^ and the sequential organ failure assessment (SOFA)^[21]^ upon intensive care unit (ICU) admission were used. Early post-CA shock within 72 hours after ROSC was defined as the need for continuous norepinephrine or epinephrine infusion to maintain mean arterial pressure >60 mmHg for >6 hours after ROSC despite adequate fluid loading.^[16]^

### 2.4 Statistical analysis

Data were analyzed using SPSS statistics for windows, version 19.0 (IBM, SPSS Inc., Armonk, NY) and MedCalc version 11.3 (MedCalc Inc., Mariakerke, Belgium). Continuous variables were presented as median and interquartile range (IQR). Meanwhile, categorical variables were presented as count and percentages unless stated otherwise. Univariate analysis was performed using the Mann–Whitney *U* test for continuous variables and the *χ*^2^ test or Fisher exact test for categorical variables.

Multivariate backward logistic regression analysis was performed to evaluate independent factors that were associated with poor neurological outcome at 3 months and mortality at 14 days and 3 months. Spearman rho (*r*) correlation coefficients were used to estimate the correlation between biomarkers and variables related to PCAS severity. To differentiate patients with poor outcomes, the test performance for PCT and S100B was calculated using the receiver-operating characteristic (ROC) analysis. The prognostic performance of the combination of PCT and S100B was assessed using the cutoff values with 100% specificity. To investigate the differences between the groups in terms of changes in the PCT and S100B levels, a linear mixed-effect model for repeated measurements was conducted with group, time, and group-by-time interaction as the fixed effects. A *P* value <.05 was considered statistically significant.

## 3 Results

### 3.1 Basic characteristics of the study populations

During the study period, a total of 128 adult OHCA survivors were treated with therapeutic hypothermia. Of these patients, 31 were excluded, of which 8 did not have consent for the study, 8 died within 24 hours, 4 were poisoned, 4 no sample, 3 had CPC **≥**3 before CA, 2 presented with cerebrovascular accident, and 2 had end-stage renal or liver disease. Finally, a total of 97 patients were included in this study.

The basic characteristics of the patients are shown in Table 1. In total, 30 patients (30.9%) had good neurological outcome, of which 27 had CPC 1 and 3 had CPC 2. A total of 67 patients (69.1%) had poor neurological outcome, of which 4 had CPC 3, 21 had CPC 4, and 42 had CPC 5 at 3 months. Thirty-five of 42 (83.3%) of patients with CPC 5 died within 14 days after ROSC.

**Table 1:**
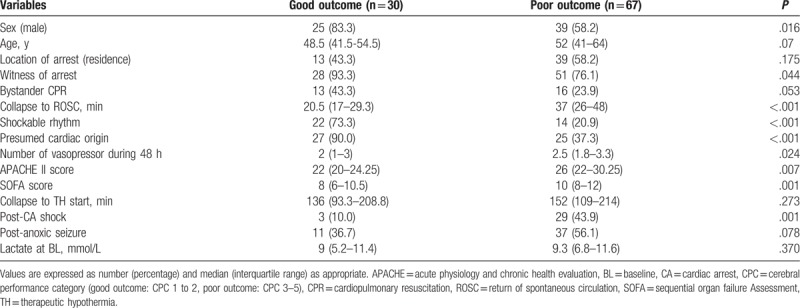
Basal characteristics of study populations.

The characteristics of patients with poor neurological outcome were as follows: female, older age, unwitnessed arrest, nonshockable rhythm, non-cardiac cause of arrest, higher incidence of post-CA shock, longer time to ROSC, higher dose of epinephrine, higher number of vasopressors, and higher SOFA and APACHE II scores.

### 3.2 Time course of PCT and S100B

**Figure 1:**
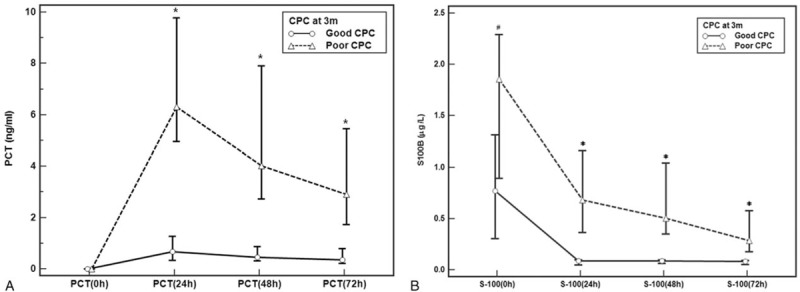
Release pattern for (A) PCT and (B) S100B protein after return of spontaneous circulation in the good and the poor outcome group. Data are presented in median and 95% confidence interval. ^#^*P* = .003, ^∗^*P* < .001. CPC = cerebral performance category, PCT = procalcitonin.

The time courses of PCT and S100B are presented in Figure 1. At BL, PCT levels cannot be differentiated. Moreover, PCT concentrations rapidly increased and peaked at 24 hours (6.32 vs 0.68 ng/mL, *P* < .001) and then decreased after 72 hours (2.9 vs 0.35 ng/mL, *P* < .001). The poor neurological outcome group had more significant changes than the good neurological group. The S100B level in both groups was highest at BL (1.86 vs 0.77 μg/L, *P* = .03) and decreased within the first 24 hours (0.68 vs 0.09 μg/L, *P* < .01), and similar levels were maintained after 72 hours (0.29 vs 0.09 μg/L, *P* < .01).

Mixed-effects model analysis showed that there was a group-by-time interaction in the PCT levels (F value = 12.606, *P* < .001) of both groups. Meanwhile, there was no group-by-time interaction in the S100B levels (F value = 1.228, *P* = .304).

### 3.3 Correlations with PCAS severity

PCT levels at 24 hours and peak levels during 72 hours after ROSC were significantly correlated with time to ROSC (*r* = 0.542/0.531), SOFA score (*r* = 0.519/0.550), APACHE II score (*r* = 0.432/0.441), CPC at 3 months (0.457/0.491), epinephrine dose used during CPR (*r* = 0.644/0.643), number of vasopressors administered within 48 hours (*r* = 0.468/0.449), and lactate levels upon admission (0.267/0.262), respectively.

S100B levels at 24 hours and peak levels during 72 hours after ROSC were significantly correlated with time to ROSC (*r* = 0.550/0.270), SOFA score (*r* = 0.259/0.223), APACHE II score (*r* = 0.320/0.348), CPC at 3 months (0.669/0.450), epinephrine dose of usage during CPR (*r* = 0.447/NS), number of vasopressors administered within 48 hours (*r* = 0.261/ 0.251), and lactate levels upon admission (0.345/0.321), respectively. PCT levels were more correlated with variables related to PCAS severity than S100B levels, whereas S100B was more correlated with brain injury than PCT.

### 3.4 ROC analysis on the prediction of poor neurological outcome

**Figure 2:**
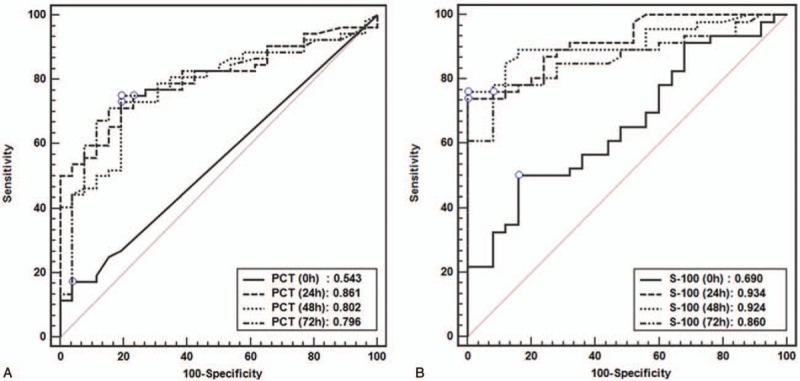
Comparison of ROC curve of PCT and S100B at 0, 24, 48, and 72 hours for predicting poor neurological outcome. (A) AUC for procalcitonin. (B) AUC for S100B protein. AUV = area under the curve, CPC = cerebral performance category, PCT = procalcitonin.

The area under the curve (AUC) of PCT and S100B levels at each time point for the prediction of poor neurological outcome are shown in Table 2, Figure 2. The most significant time point with the highest AUC value (0.861) of PCT was at 24 hours, and the cutoff value, sensitivity, and specificity at 24 hours were 2.62 ng/ml, 70.2%, and 83.3%, respectively. The 100% specific cutoff values of PCT level for the prediction of a poor neurological outcome at BL, 24, 48, and 72 hours were 0.53, 6.52, 6.96, and 17.07 ng/mL, respectively.

**Table 2:**
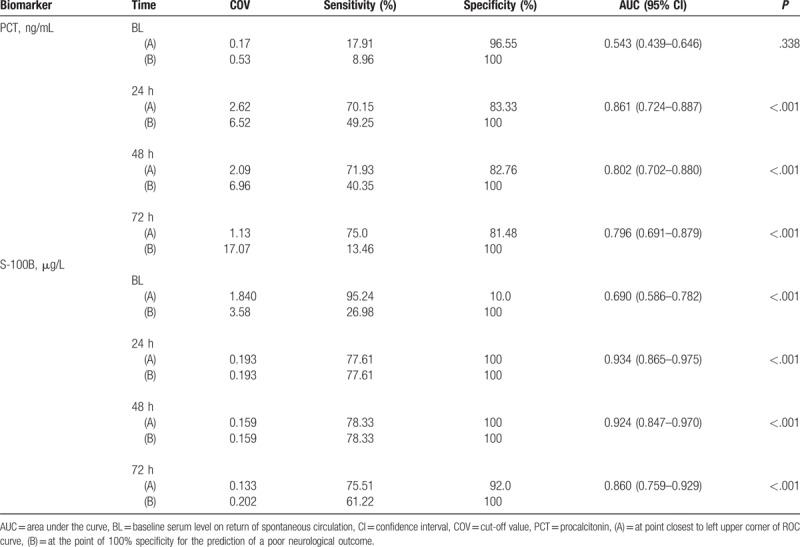
ROC analysis of PCT and S100B protein level for prediction of poor neurological outcome.

The most significant time point with the highest AUC value of S100B was 24 hours, and the cutoff value, sensitivity, and specificity at 24 hours were 0.193, 77.6%, and 100%, respectively. The 100% specific cutoff values of S100B for the prediction of poor neurological outcome at BL, 24, 48, and 72 hours were 3.58, 0.193, 0.159, and 0.202 μg/L, respectively.

### 3.5 Combination of S100B and PCT

**Figure 3:**
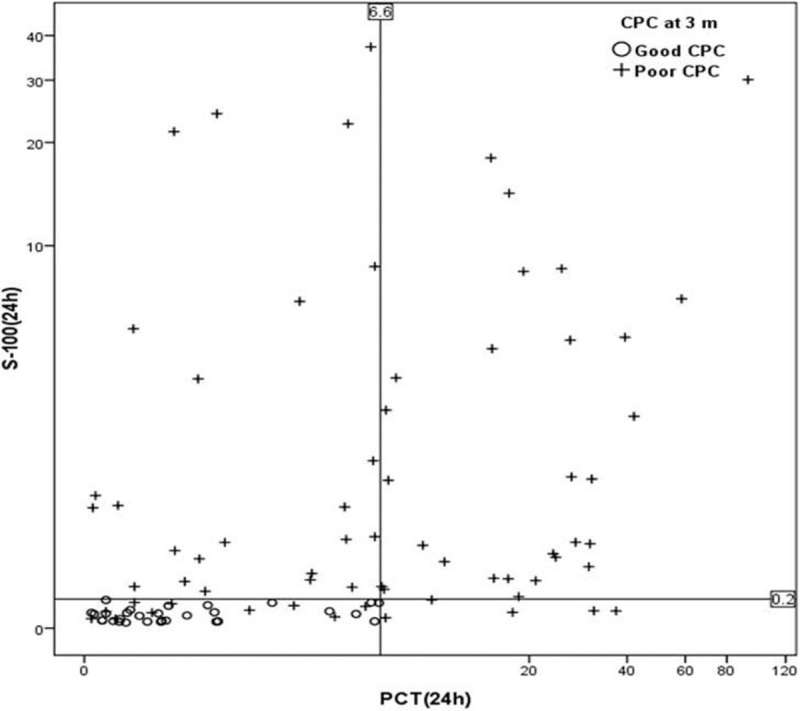
A scatter plot depicting the relationship between PCT and S100B protein according neurological outcome at 24 hours after return of spontaneous circulation. Line indicates 100% specific cutoff values of PCT (6.6 ng/mL) and S100B protein (0.2 μg/L). PCT = procalcitonin.

The combination of 24-hour S100B and PCT (S100B level ≥0.2 μg/L or PCT level ≥6.6 ng/mL), wherein a poor neurologic outcome is predicted if at least one of the 2 tests indicates poor outcome, has a higher sensitivity in predicting poor neurological outcome than S100B alone (77.61% [95% confidence interval, CI: 67.4–88.1] vs 85.07% [95% CI: 74.3–92.6]) with 100% specificity (Fig. 3).

### 3.6 Multivariate logistic regression analysis of mortality and neurological outcome

In the multivariate logistic regression analysis, age, sex, noncardiac origin, time to ROSC, and 24-hour S100B (odd ratio [OR]: 9.849, 95% CI: 2.089–46.431, per 0.1) were independently associated with poor neurological outcome at 3 months, whereas 24-hour PCT was not. However, if 24-hour PCT alone, without S100B, is used as a covariate in the analysis, 24-hour PCT (OR: 1.656, 95% CI: 1.129–2.427) was found to be associated with poor neurological outcome (Table 3).

**Table 3:**
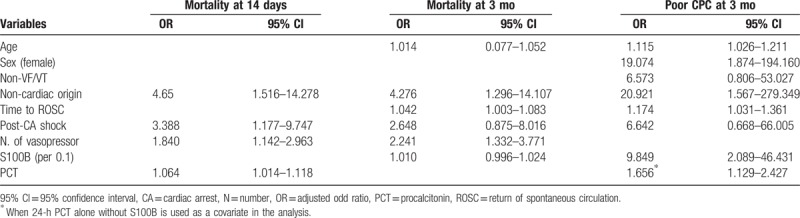
Multivariate logistic regression analysis.

Noncardiac origin, post-CA shock, number of vasopressors administered within 48 hours, and 24-hour PCT (OR: 1.064, 95% CI: 1.014–1.118) were independently associated with 14-day mortality, whereas S100B was not. However, both 24-hour PCT and S100B were not associated with 3-month mortality.

## 4 Discussion

In this prospective observational study, serum PCT and S100B levels were found to be significantly associated with neurological outcome, and both have good discriminative power for the prediction of poor neurological outcome in post-CA patients. In addition, the combination of the 2 biomarkers had a better prognostic performance, particularly sensitivity, than the use of either biomarker alone. To the best of our knowledge, this is the first published study on the use of the combined 2 biomarkers for the prognostication of post-CA patients. These findings indicate that S100B and PCT might play a specific role in the prognostication of PCAS.

The poor outcome observed in post-CA patients is attributed to a unique pathophysiological process involving multiple organs termed the PCAS,^[22]^ defined as post-CA brain injury, myocardial dysfunction, and systemic ischemic–reperfusion response. Although majority of post-CA patients died because of brain injury, MOD and cardiovascular instability or post-CA shock are common and are associated with a significant proportion of death from PCAS. Laver et al and Lemiale et al^[12,14]^ have reported that post-CA shock and MOD accounted for approximately 30% to 45% of deaths, and shock-related death occurred during the early stage, mostly within 14 days after ICU admission. Therefore, post-CA shock and MOD were among the major causes of early death, and biomarkers, such as PCT, might be useful for the prediction of poor outcome. PCT is a 116-amino-acid peptide secreted from the C-cells of the thyroid and a biomarker of systemic inflammatory response.^[23]^ PCT has been shown to be a valuable marker to detect bacterial infection, guide antibiotic therapy, and predict outcome in patients with pneumonia and other causes of severe sepsis.^[24,25]^ Recently, an elevated PCT level occurs not only in patients with infection but also in critically ill patients with various causes.^[26,27]^

Several studies have demonstrated that early PCT levels represent an inflammatory response of PCAS and are associated with the severity of PCAS and long-term outcome. Moreover, it has been found^[17]^ that early PCT levels are significantly correlated with time to ROSC, a surrogate marker for the duration of ischemia and the SOFA score, which is a clinical score that quantifies the severity of PCAS. In other studies, elevated PCT levels were found in patients with cardiogenic shock^[28]^ or post-resuscitation shock and those who were on renal replacement therapy.^[29]^

Our results also showed that 24-hour PCT levels were correlated with time to ROSC (*r* = 0.542), SOFA score (*r* = 0.519), epinephrine dose (*r* = 0.644), number of vasopressors administered within 48 h (0.468), short-term mortality (14 days) (OR: 1.064, 95% CI: 1.014–1.118), and poor CPC after 3 months (OR: 1.656, 95% CI: 1.129–2.427). These results indicated that PCT is more likely to reflect the severity of ischemic–reperfusion injury, which results in severe MOD and ultimately death. Therefore, PCT might be a strong indicator of the severity of PCAS during the early post-CA period and PCT may be one of the useful candidates in the multimodal approach for the prognostication of post-CA patients. However, the evidence is still insufficient and more researches are required.

With regard to neurological outcome prediction, our results (AUC: 0.80–0.86) are similar to those of previous studies,^[17,30]^ with TH ranging from 0.84 to 0.89. Although some studies showed AUC >0.9, even higher than that of S100B,^[4,31]^ their participants partly received or did not receive MTH at all, and the sample size of these studies are small. The cutoff values and test performance for PCT varied, and the clinically useful thresholds were not identified. This may be because of the different characteristics of the study participants (eg, initial rhythm, cause of arrest, TH application, hypoxic time, and poor outcome ratio) and heterogeneous measurement methods^[1]^ between studies. However, previous studies^[4,17,31]^ had the highest PCT levels and the most effective prognostic performance at 24 or 48 hours, which is similar to the results of our study.

The S-100B protein is a calcium-binding protein with a molecular weight of 21 kDa and a half-life of 2 hours, which is expressed mainly in astroglial cells.^[32]^ Increased serum levels of S100B protein might cause neuronal apoptosis^[33]^ and has been reported during the acute phase of brain damage in the various conditions.^[34]^ Therefore, it has been found to be an early and sensitive marker of hypoxic brain damage and neurological outcome after CA.^[3–11]^

With regard to neurological outcome prediction, the blood values of S100B after CA are likely correlated with the extent of anoxic–ischemic neurological injury from CA and with the severity of neurological outcome. Although several studies^[3,7,9]^ had high AUC (0.9–1.0) and a similar cut off value (0.18–0.21) with our results for the prediction of poor neurological outcome, other studies^[6,10,35]^ have shown a limited value and variability of thresholds. However, previous studies^[3,9,10]^ have shown that S100B levels at 24 hours had the highest discriminative power for the prediction of poor outcome, which is similar to the results of our study. This is one of the reasons why we choose 24 hours as the time point of the combination for outcome prediction.

Stammet et al^[35]^ have evaluated the effectiveness of the combination of S100 and NSE. However, they have failed to demonstrate any improvement in outcome prediction. This may be because of similar and overlapping characteristics as an indicator of brain injury. Airborn et al^[15]^ have found that the combination of NSE and MR-proANP or TnT improved diagnostic accuracy, whereas the biomarkers of endogenous stress (CT-proAVP) or inflammation (PCT) marginally improved or did not at all improve prognostic accuracy. However, they have suggested the need of a brain-specific biomarker with more earlier release profile, such as S-100B protein, for the earlier prognostication of outcome after CA. Fries et al have first reported that the prognostic value of PCT was higher than that of S100B for the outcome prediction in post-CA patients who were not treated with TH. However, they did not try a combination of 2 biomarkers for outcome prediction. Thus, our study found the combination of 24-hours S100B and PCT (S100B ≥0.2 μg/L, or PCT ≥6.6 ng/mL) that improved the sensitivity in predicting poor neurological outcome compared to S100B alone (77.61% [95% CI: 67.4–88.1] vs 85.07% [95% CI: 74.3–92.6]) with 100% specificity. During the early stage of post-resuscitation period, a considerable number of patients may die because of cardiovascular shock and MOD without severe brain injury. These patients may not have elevated brain-specific biomarkers that cause the decreased accuracy of the biomarkers. Therefore, the added value of the PCT measurement was used to identify few but relevant false-negative patients with low S100B level but poor outcome. Our results suggested that early elevation of PCT levels after CA plays a role in the early identification of patients who are at higher risk for circulatory failure, MOD, and even death, and more aggressive and intensive treatments can be provided to these patients.

In the multivariate analysis of our study, PCT was found to be independently associated with mortality at 14 days, whereas S100B was associated with poor neurological outcome at 3 months. In addition, neither biomarker was associated with 3-month mortality. These results may indicate the different role of these biomarkers for the prognostication of post-CA patients, which increases because of different release mechanisms, of which the elevation of PCT is owing to extra-cerebral organ injury, such as shock and MODS, and that of S100 is attributed to cerebral injury. Hence, PCT may be useful in predicting early mortality, whereas S100B may be useful in predicting late neurological outcome.

This study has several limitations. The cutoff value in our study is difficult to apply in clinical settings because of a small sample size at a singer center. However, we demonstrated the specific role of each biomarker according to different post-resuscitation periods in predicting outcome.

The current neurologic outcome prediction of comatose CA survivors focuses on the end-of-life decision, such as the withdrawal of life-sustaining therapy (WLST). In Korea, WLST is not permitted unless the patient is brain dead and donates organs. Therefore, resuscitated comatose patients still receive intensive care support for prolonged periods compared with patients in Europe^[36]^ and this may lead to different outcomes during the early stage in other studies. The difference between patient characteristics, such as relatively lower percentage of patients with good neurological outcome, shockable rhythm, and presumed cardiac origin, can cause some difficulties in comparing the results of previous studies.

## 5 Conclusions

The results of this study support the assumption that PCT is associated with extracerebral organ dysfunction and subsequently early mortality during the post-resuscitation period, whereas S100B was associated with brain injury and late neurological outcome in CA patients. Therefore, the combination of the 2 biomarkers might improve prognostic performance, particularly sensitivity, compared to the use of either biomarker alone. However, further studies that will identify the optimal cutoff values for these biomarkers must be conducted.

## Author contributions

**Conceptualization:** Jae Ho Jang, Won Bin Park, Yong Su Lim.

**Data curation:** Jae Ho Jang, Won Bin Park, Jea Yeon Choi, Jin Seong Cho, Woo Sung Choi.

**Formal analysis:** Jae Ho Jang, Won Bin Park, Yong Su Lim, Jin Seong Cho.

**Investigation:** Jea Yeon Choi, Jae-Hyug Woo, Woo Sung Choi.

**Methodology:** Jae Ho Jang, Won Bin Park, Yong Su Lim.

**Project administration:** Yong Su Lim.

**Supervision:** Yong Su Lim, Jin Seong Cho, Jae-Hyug Woo, Hyuk Jun Yang, Sung Youl Hyun.

**Validation:** Yong Su Lim, Jea Yeon Choi, Jin Seong Cho.

**Visualization:** Jae-Hyug Woo.

**Writing – original draft:** Jae Ho Jang, Won Bin Park, Yong Su Lim.

**Writing – review & editing:** Yong Su Lim.

Yong Su Lim orcid: 0000-0003-4390-4010.
